# From uni- to multimodality: towards an integrative view on anuran communication

**DOI:** 10.1007/s00359-014-0923-1

**Published:** 2014-06-29

**Authors:** Iris Starnberger, Doris Preininger, Walter Hödl

**Affiliations:** 1 Department of Integrative Zoology, University of Vienna, Althanstraße 14, 1090 Vienna, Austria; 2 Vienna Zoo, Maxingstraße 13b, 1130 Vienna, Austria

**Keywords:** Bioacoustics, Visual signals, Chemical signals, Frogs, Toads, Signal modalities

## Abstract

Undeniably, acoustic signals are the predominant mode of communication in frogs and toads. Acoustically active species are found throughout the vast diversity of anuran families. However, additional or alternative signal modalities have gained increasing attention. In several anurans, seismic, visual and chemical communications have convergently evolved due to ecological constraints such as noisy environments. The production of a visual cue, like the inevitably moving vocal sac of acoustically advertising males, is emphasized by conspicuously coloured throats. Limb movements accompanied by dynamic displays of bright colours are additional examples of striking visual signals independent of vocalizations. In some multimodal anuran communication systems, the acoustic component acts as an alert signal, which alters the receiver attention to the following visual display. Recent findings of colourful glands on vocal sacs, producing volatile species-specific scent bouquets suggest the possibility of integration of acoustic, visual and chemical cues in species recognition and mate choice. The combination of signal components facilitates a broadened display repertoire in challenging environmental conditions. Thus, the complexity of the communication systems of frogs and toads may have been underestimated.

## Introduction

A great diversity of signalling strategies and behaviours can be observed during animal communication shaped by sexual selection and the environmental constraints (Narins and Zelick 1988; Endler 1992; Endler and Thery 1996; Leal and Fleishman 2004; Bradbury and Vehrencamp 2011). In several species, not only one communication mode, but two or more are used simultaneously or sequentially across multiple sensory components (Partan and Marler 1999; reviewed in Candolin 2003; Hebets and Papaj 2005; Otovic and Partan 2009). Multimodal communication is discussed for a wide range of species including spiders (e.g. Uetz et al. 2009), fish (e.g. Van Staaden and Smith 2011), reptiles (reviewed in Hews and Martins 2013), birds (e.g. Wiley 1973) and mammals (e.g. Bro-Jorgensen and Dabelsteen 2008), but has been difficult to test until recently. Complex signalling repertoires are challenging to investigate and valid hypotheses testing remains difficult in scientific experiments (Leger 1993; Partan and Marler 2005; Rosenthal 2007). However, advances in conceptual framework and technical equipment have greatly improved research in this field. In anuran amphibians, calls are the predominant signals in inter- and intrasexual communication (Ryan 1985; Gerhardt and Huber 2002; Dorcas et al. 2010). Vocalizations are the most conspicuous display to human observers and as a consequence other signal modalities have traditionally received less attention (Waldman and Bishop 2004; Coleman 2009) or may have been misjudged, e.g. due to experimenters’ lack of visual sensitivity at night (Buchanan 1993). Amphibians in general and frogs in particular are excellent model organisms to experimentally investigate communication strategies both in the laboratory and under natural conditions, as they are hardly disturbed by observers and can be easily manipulated (Narins et al. 2003; Hirschmann and Hödl 2006; Taylor et al. 2007).

This review highlights anuran signalling strategies in addition to calling to promote an integrative multimodal view on anuran communication.

## Anurans are born to call

“Frogs enjoy life and express their joy by song” (Dickerson 1908). Much has changed since these lines were written, and due to numerous studies on acoustic signals in anurans, we have a very different albeit less romantic opinion on signal content and function. The male advertisement call attracts conspecific females and signals the readiness to defend territories and calling sites to rival males; hence calling behaviour plays a vital role in reproductive success and is essential for sexual selection (Narins et al. 2007). Many frogs and toads have more than one species-specific call type. In addition to the prominent advertisement call a variety of discrete or continuous call types correspond to specific functions, such as the encounter call (McDiarmid and Adler 1974), the courtship call, the territorial call, the distress call, and the release call (all reviewed in Wells 1977).

### How frogs and toads get their acoustic message across

In numerous species, audio-spectral and temporal call characteristics and their function as static or dynamic signal properties were investigated during the last decades (Gerhardt and Huber 2002). Robert R. Capranica was the first to combine electrophysiological analyses, behavioural data and synthetic playback calls to study “what the frog’s ear tells the frog’s brain” (Capranica and Moffat 1983; and see Simmons 2012). As a mentor, he inspired future generations to study animal communication by means of integrative research in the lab and also in the field and opened-up a new field which likewise attracted bioacousticians and evolutionary biologists. As a consequence, anuran vocal signals and their perception are nowadays an exceptionally well-understood subject in biology. Anuran call characteristics correlate with body size and mass across species (Ryan 1988; Gingras et al. 2013) and within species (e.g. Narins and Smith 1986; Robertson 1990) a pattern described as the Deep croak hypothesis by Davies and Halliday (1979); and see Gingras et al. (2013). Furthermore, call parameters signal species identity (e.g. Blair 1958; Hödl 1977), and in some species exhibit “individual” distinctive signatures (Bee and Gerhardt 2001a, b; Gasser et al. 2009), but not in others (Bee 2003). Advertisement calls also regulate male spacing (e.g. Brenowitz 1989), increase the male’s attractiveness to females (e.g. Ryan and Keddy-Hector 1992) and have evolved to match the tuning of the receiver’s auditory system (Gerhardt and Schwarz 2001).

Any message needs to be successfully transmitted to elicit the intended response in the receiver (Shannon 1948); clear reception is a minimum requirement for a successful communication system (Shannon 1948; Endler 1993). The most basic requirement for a call is to be detectable against background noise and to minimize transmission degradation and attenuation of the environment. High levels of biotic and abiotic environmental noise may mask calls and hamper accurate detection, discrimination and localization (or increase response latency) by receivers (Bee 2008; Vélez et al. 2012, 2013; Caldwell and Bee 2014). However, signal properties and strategies have been shaped over evolutionary time to enhance transmission in their respective acoustic environments by preferences of receivers.

**Fig. 1:**
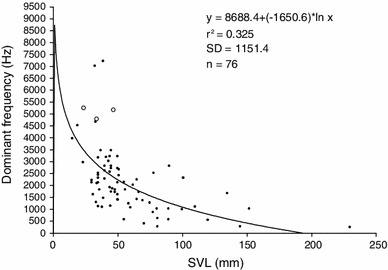
Logarithmic regression of call frequency on body size of 76 male ranid frogs. *Circles* denote foot-flagging species of the stream-associated genus *Staurois* (*S. parvus*, *left*; *S. guttatus*, *middle*; *S. latopalmatus*, *right*) emitting higher pitched calls than other ranids of similar body size. Graph was adapted with permission of the authors, for detailed data on the species see (Boeckle et al. 2009)

To improve signalling effectiveness, anuran species organize calling periods temporally (Klump and Gerhardt 1992), adjust their calls to random intercall intervals (Zelick and Narins 1985) or inhibit calling heterospecifics (Schwartz and Wells 1983). In dense breeding aggregations, concurrently chorusing conspecifics can achieve release from masking interference by spatial separation from the biotic sound source (Grafe 1996; Bee 2008). In the presence of continuous background noise, the recognition and detection of acoustic signals can be impaired dependent on the relative noise level and frequency (Feng and Schul 2006). In torrent frogs, high-frequency and in some cases even ultrasonic calls enhance the signal-to-noise ratio relative to low-frequency stream noise and are suggested to be an adaptive strategy in the presence of continuous noise (Narins et al. 2004; Feng et al. 2006; Boeckle et al. 2009). For example, stream-associated species of the genus *Staurois* emit higher pitched calls than other ranid species of comparable body size (Fig. 1).

Acoustic adaptations to overcome signalling constraints are limited and often opposed by morphological and phylogenetic constraints—but most importantly by sexual selection (e.g. Ryan and Rand 2003). In many cases, species develop new, often spectacular ways of communication in which the production of acoustic signals is of less importance or even completely abandoned (Rödel et al. 2003; Hirschmann and Hödl 2006; Preininger et al. 2009).

## When calling is not enough: from single to multimodality

“Compared to salamanders, olfactory cues and visual displays seem to be unimportant to preamplectic courtship in most anurans, but some tactile cues are used by certain species.” Duellman and Trueb (1986) wrote in their widely used textbook “Biology of Amphibians” published almost 30 years ago. Today numerous new findings in several anuran species in regard to colourations, visual displays and even olfactory cues utilized as signals in conspecific communication depict a somewhat different story.

### The role of the vocal sac in communication

The anuran vocal sac likely evolved in response to selection for increased calling efficiency (Bucher et al. 1982; Pauly et al. 2006). It also minimizes the loss of sound energy by decreasing the impedance mismatch between the frog’s body cavity and its environment, increases the call rate and distributes sound waves omnidirectionally (Bucher et al. 1982; Rand and Dudley 1993; Pauly et al. 2006). Colour and shape of the vocal sac display were probably first incorporated as a visual cue and selection pressures later shaped various conspicuous signal variations. The visual component of the vocal sac increases detection through movement and colouration (Rosenthal et al. 2004; Taylor et al. 2008), thereby enhancing the call attractiveness to females and aggression during territorial male–male interactions. Females of the Túngara frog (*Engystomops pustulosus*) prefer advertisement calls in addition to the visual cue of a pulsating vocal sac over the call alone under low sound pressure levels. However, when the visual stimulus is presented with a less attractive slow call rate, females rather choose the attractive unimodal call, which emphasizes that vocalizations are necessary for mate attraction (Taylor et al. 2011a). Comparisons of unimodal acoustic stimuli to multimodal stimuli presentations in the Kottigehar Dancing Frog (*Micrixalus kottigeharensis*; former identification as Small Torrent Frog (*Micrixalus* aff. *saxicola*)) demonstrated that a pulsating white vocal sac increases the frequency of response behaviours in conspecific males and elicits an agonistic visual display response (Preininger et al. 2013c). The visual cue of the vocal sac was suggested to mainly facilitate detection and localization of mating partners or opponents in noisy choruses (Rosenthal et al. 2004; Taylor et al. 2008, 2011b; Preininger et al. 2013a).

**Fig. 2:**
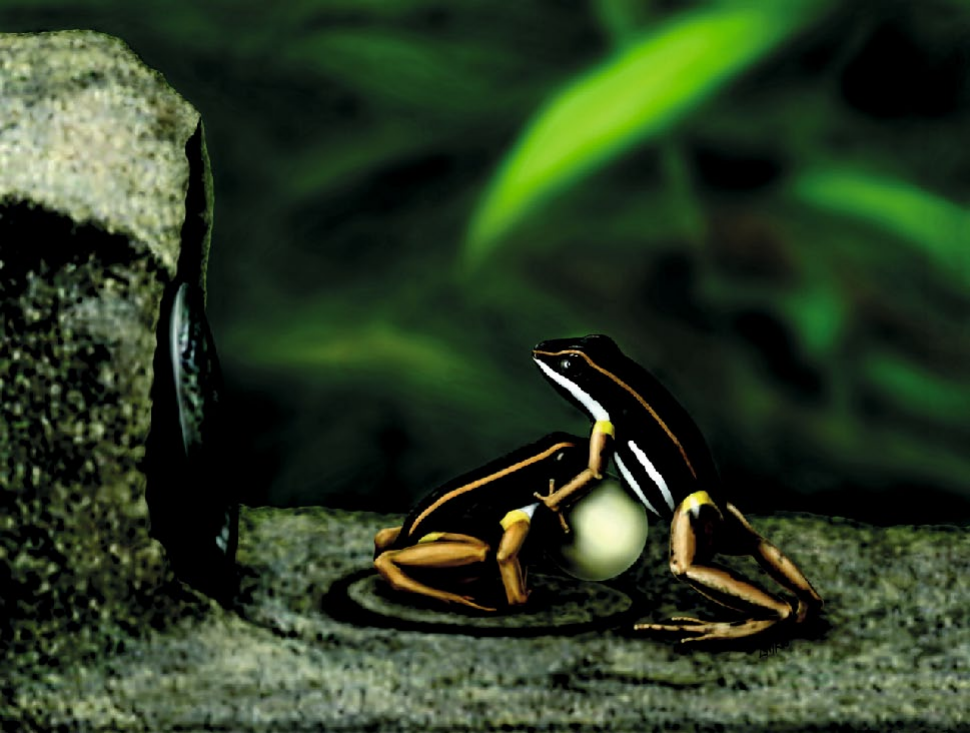
A rendering from video frame illustrating aggressive (fighting) behaviour of an *A. femoralis* male (*right*) toward the electromechanical model frog (*left*) placed in his territory, 2 m from his initial calling position. In all experimental trials that evoked fighting, the model’s vocal sac was inflated and pulsating, and was accompanied by playback of the male’s species-specific territorial call. With kind permission: Narins et al. (2003), Fig. 3. Copyright (2003) National Academy of Sciences, USA

The first evidence for the role of a pulsating vocal sac as a visual cue during male territorial defense comes from studies in the brilliant thighed dart-poison frog *Allobates femoralis*. No unimodal stimulus (call or pulsating vocal sac) was able to elicit territorial aggression in the opponent male, only temporally overlapping dynamic bimodal cues evoked fighting behaviour (Narins et al. 2003, 2005). Hence, conspecific vocalizations in *A. femoralis* trigger a phonotactic response and antiphonal calling but are not sufficient to evoke physical aggression (Fig. 2). De Luna et al. (2010) showed that the movement of the inflated grey vocal sac itself was not important, but movement of the frog dummy per se (i.e. jumping) evoked territorial aggression in *A. femoralis* males. Conspicuous colourations, however, are often crucial in visual vocal sac displays, such as in the East-African stream frog *Phrynobatrachus kreffti* where conspicuous yellow vocal sacs function as signals in male–male agonistic interactions even without calls being emitted (Hirschmann and Hödl 2006).

The inevitable movement of the vocal sac during sound production can also act as vibrational or seismic cue. In the mostly ground-dwelling white-lipped frog *Leptodactylus albilabris* vocal sac inflations against the ground produce substrate-borne vibrations (Lewis et al. 2001) which act as additional seismic signals to overcome heterospecific chorus noise (and see Cardoso and Heyer 1995). Frogs and toads calling in the water produce circular waves travelling from the calling individual. It has been shown in the lateral line bearing Fire-bellied toad *Bombina bombina*, that individuals floating in the vicinity of the caller perceive this water movement and respond to it (Seidel et al. 2001). When males of the nocturnal Red-eyed tree frog (*Agalychnis callidryas*) perceive plant-borne vibrations indicating a conspecific intruder, they in turn shake branches as an agonistic display (Caldwell et al. 2010). The tremulations of *A. callidryas* correlate with individual dominance and might play a role in sexual selection, comparable to seismic signals in wolf spiders (Elias et al. 2005), moreover, they constitute a signalling modality dissociated from vocalizations.

### Visual signals are often more than just a byproduct of calling

**Fig. 3:**
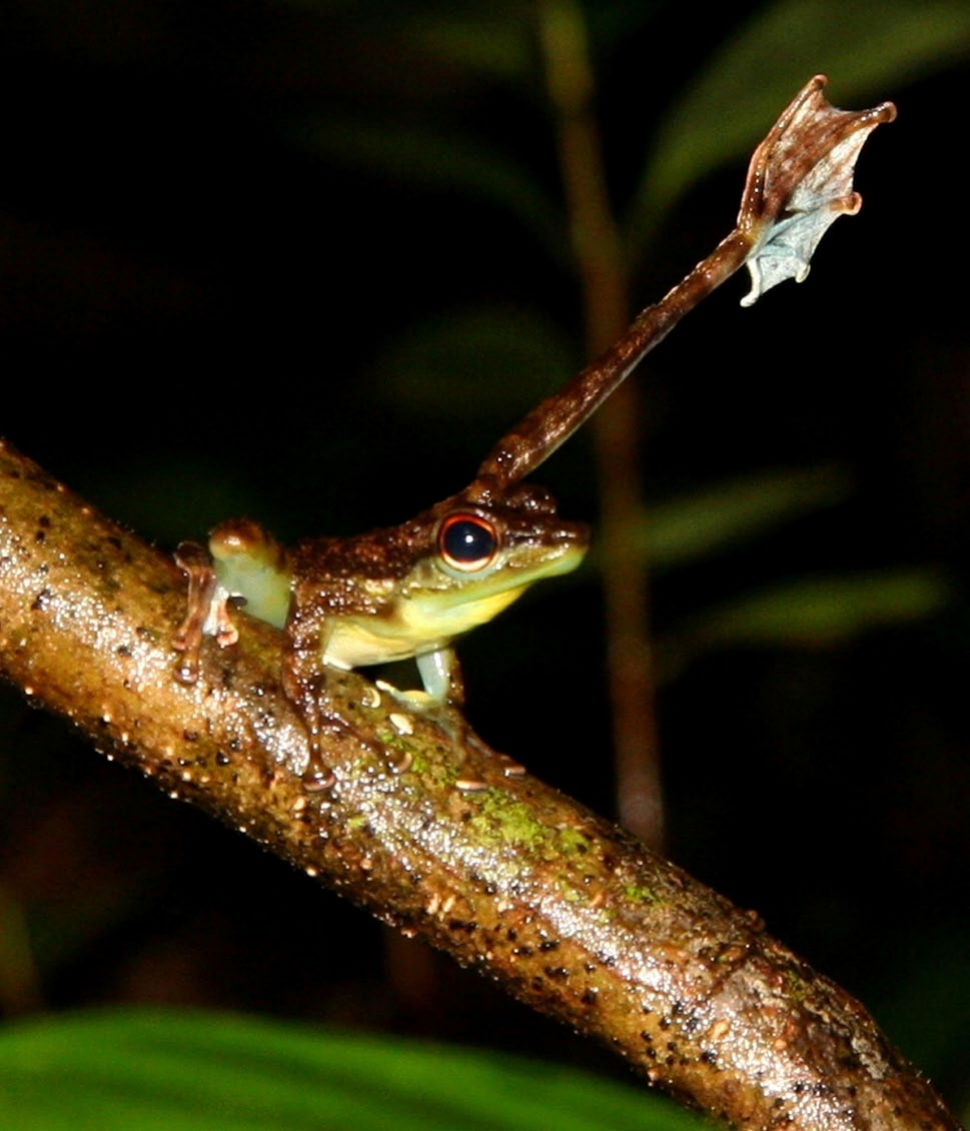
Male *Staurois guttatus* performing the agonistic visual signal termed “foot flagging” with his *left* hind leg

Visual displays which can be presented independently of acoustic signals have been observed in several anuran species. Limb movements are used in addition to acoustic signals during courtship and male–male interaction (reviewed in Hödl and Amézquita 2001; Hartmann et al. 2005). The most striking visual signalling behaviour to the human observer is foot flagging, a display during which the hind leg is raised, the toes are spread and conspicuously coloured interdigital webbings are displayed (Fig. 3). Foot flagging has been reported in 16 anuran species from five different families (Hödl and Amézquita 2001; Vasudevan 2001; Hartmann et al. 2005; Krishna and Krishna 2006; Grafe and Wanger 2007). The signalling behaviour is mainly known from diurnal, stream-dwelling species (but see Amézquita and Hödl 2004) and is displayed predominantly during male–male interaction or territorial encounters (Hödl 1977; Hödl et al. 1997; Haddad and Giaretta 1999; Hödl and Amézquita 2001). In the Bornean genus *Staurois*, foot-flagging behaviour is suggested to function as an additional or alternative mode of communication in noisy stream environments. The high pitched calls of *S. guttatus*, *S. latopalmatus* and *S. parvus* are thought to alert receivers and direct their attention to the subsequent visual signal (Grafe and Wanger 2007; Preininger et al. 2009; Grafe et al. 2012). The functional separation of acoustic and visual signals allows to study influences of signal components on receivers without a determining linkage of signal modality and/or adaptation. Foot-flagging displays in the Kottigehar Dancing Frog (*M. kottigeharensis*) do not form fixed-composite signals (sensu Partan and Marler 2005) with vocalizations. Preininger et al. (2013c) suggest that the conspicuous display is a ritualization of physical attacks. Agonistic foot-flagging signals might have developed to minimize leg-kicks, a fighting technique predominantly observed in aggressive close-range encounters (Preininger et al. 2013c) and comparable to intimidation displays in bushbucks (Wronski et al. 2006; and see Smith and Evans 2011). Reactions to components of multimodal signals showed differing influences on conspecific receivers in across-species comparisons of foot-flagging species (Preininger et al. 2013b). Different signal function of multiple signal components in female mate preference was also demonstrated in squirrel tree frogs (*Hyla squirella*) and Túngara frogs (*E. pustulosus*) (Taylor et al. 2011b).

A further example of visual signalling behaviour shaped by intrasexual selection was demonstrated in recent investigations of sexual dichromatism in explosively breeding Moor frogs (*Rana arvalis*). The temporal blue nuptial colouration in males was suggested to be a visual signal promoting instantaneous mate recognition and allowing males to quickly move between rivals while scrambling for females (Ries et al. 2008; Sztatecsny et al. 2010, 2012). Nuptial dichromatism was reported for at least 31 species from seven families (Hoffmann and Blouin 2000; Bell and Zamudio 2012), however, the function of this spectacular phenomenon remains largely unexplored for most species. Hence, successful communication strategies in noisy environments can be developed in the acoustic or visual domain, if they are beneficial for sender and receiver (reviewed in Brumm and Slabbekoorn 2005).

Visual signals in combination with or instead of acoustic signals already broaden an anuran’s signal repertoire drastically, but evidence is emerging that some species utilize additional modalities.

### Adding chemicals to the signal cocktail

A wide range of aquatic and terrestrial amphibians use chemical cues for orientation (Sinsch 1990; Schulte et al. 2011), prey detection (Shinn and Dole 1978; David and Jaeger 1981; Dole et al. 1981) and predator detection (Flowers and Graves 1997), which leads to the assumption that many species may have the physiological and anatomical abilities to produce and detect hetero- and conspecific chemical signals (Byrne and Keogh 2007; Woodley 2010; Hamer et al. 2011).

In aquatic and terrestrial urodeles, there are several well-known cases of chemical communication in a sexual context, such as in newts of the genus *Triturus* (Malacarne and Giacoma 1986) where chemical signals may even be more important than visual signals (Treer et al. 2013). There are few reported cases of pheromones in aquatic anurans. Females of the Magnificent tree frog (*Litoria splendida*) are attracted to the male by “splendipherin”, an aquatic pheromone produced by males in glands on the head (Wabnitz et al. 1999). In African clawed-frogs (*Hymenochirus* sp.), females tested in Y-maze experiments showed a clear preference for water containing homogenized male post-axillary breeding glands or water previously containing live males (Pearl et al. 2000). The chemicals found in *L. splendida* and in *Hymenochirus* sp. are non-volatile peptides and can, therefore, only be spread in water (Rajchard 2005; Houck 2009).

A considerable number of publications speculate about the use of skin glands present in males of many terrestrial anurans with regard to chemical communication in a sexual context due to their direct contact with the female during amplexus (Thomas et al. 1993; Rödel et al. 2003; Lenzi-Mattos et al. 2005; Willaert et al. 2013). However, only two cases of pheromone signals in terrestrial amphibians are presently reported. In the Australian toadlet *Pseudophryne bibronii*, males call hidden in the leaf litter at night and secrete an odorous mucus produced by dorsal, axillary and postfemoral skin glands, which is likely to aid females in close-range mate localization and significantly influences male calling activity (Byrne and Keogh 2007). In mantellid frogs native to Madagascar, males have prominent femoral glands, which produce volatiles possibly acting as species-specific pheromones (Poth et al. 2012).

**Fig. 4:**
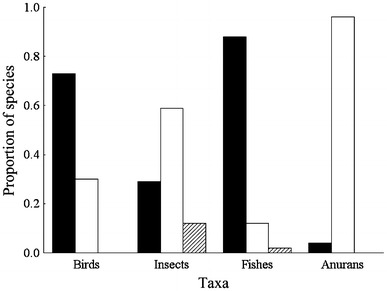
Proportion of species studied within the most well-represented taxa found to use one or more of the most frequently investigated sensory modalities in mate choice (visual: *solid bars*; acoustic: *open bars*; chemical: *cross-hatched bars*). With kind permission from Coleman (2009), Fig. 2

The use of pheromones in anuran species recognition and mate choice might be a widespread phenomenon, since chemosignals can usually be produced at low costs (Hedin et al. 1974). To date, chemical communication in anurans has been overlooked by most studies (Waldman and Bishop 2004; Belanger and Corkum 2009) in contrast to the vast number of studies on chemical communication in caudate amphibians (e.g. Vaccaro et al. 2010; Treer et al. 2013), mammals (e.g. Johnston et al. 1999) or insects (Hölldobler and Wilson 1990; Carde and Minks 1997). There is a strong disparity in the number of studies on different signal modalities in several taxa which might have led to a biased view on their signalling repertoire (Fig. 4).

**Fig. 5:**
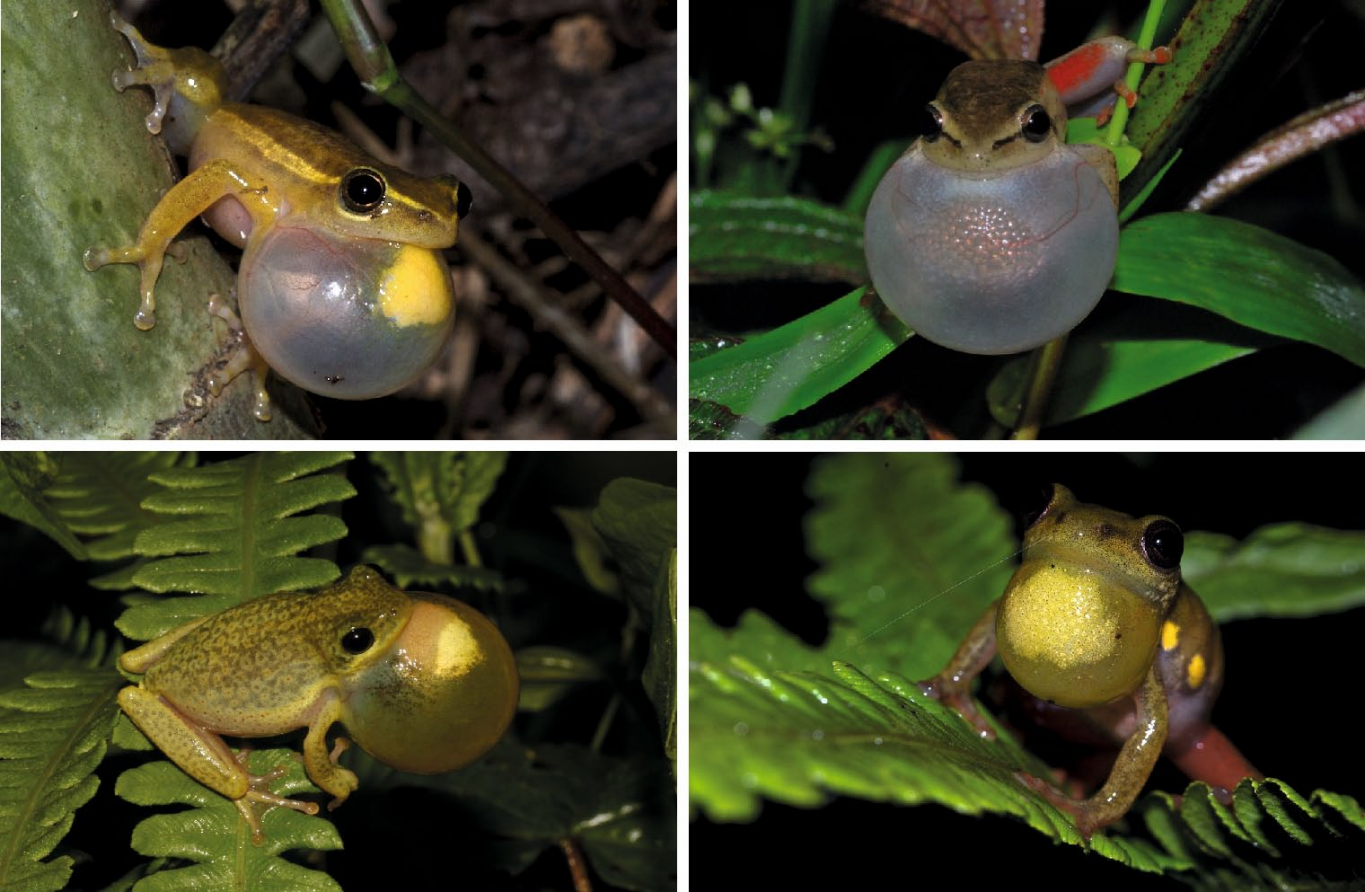
(From *top left* to *bottom right*): Sympatric male individuals of *Hyperolius cinnamomeoventris*, *H*. *kivuensis*, *H*. *viridiflavus* and *H. lateralis* with inflated vocal sac. The prominent gular patch is visible in all pictures. (Photos by I. Starnberger and W. Hödl, all taken at Kibale Forest National Park, Uganda). With kind permission: Starnberger et al. (2013), Fig. 1. Copyright (2013) The Authors and The Linnean Society of London, UK

The first report of possible trimodal communication in anurans comes from the species-rich frog family Hyperoliidae (Starnberger et al. 2013). Within this clade there is substantial variation in colouration, morphology, and reproductive modes, but males of most reed frog species share a common feature: a prominent gular gland on the vocal sac (Fig. 5). Chemical cocktails found in the gular gland are species specific and a combination of acoustic and chemical signals is most likely used to enhance detectability of conspecifics within dense multi-species breeding aggregations typical for hyperoliid frogs. The conspicuously coloured vocal sacs could add a further visual signalling component to the display. Hence, the vocal sac could act as a trimodal signal source which simultaneously emits acoustic, visual and chemical signals to facilitate detection, discrimination and/or location in conspecific receivers.

To understand uni- or multimodal signal efficacy we also have to question how effectively a signal influences the receiver’s sensory system and subsequently its behaviour. Sophisticated experiments are needed to test, whether the signal must be trimodal to elicit a behavioural reaction in the receiver.

### The receiving end of anuran communication

Sensory perception of any signalling modality is mediated by receptors of the receiver sensory system. Mechano-, photo- and chemoreceptors react to signal stimuli and transduce the signal into electrical impulses processed by the nervous system. Acoustic vibrations of a frog’s call are transmitted to two sensory organs of the inner ear specialized for the reception of sound (reviewed in Capranica 1976; Narins et al. 2007). The amphibian papilla is tonotopically organized and reacts to frequencies ranging between 80 and 1,600 Hz from the rostral to the caudal part respectively, whereas the non-tonotopic basilar papilla is sensitive to frequencies above 1,600 Hz (Vélez et al. 2013). Colour and brightness, hence wavelength and intensity of perceived light, of a visual signal stimulus are processed by photoreceptors in the retina. Like most vertebrates, frogs and toads possess two types of photoreceptors—rods and cones (Kelber and Roth 2006). The anuran retina typically contains two spectral classes of rods, sensitive to the intensity of light and three spectral classes of cones, responding to colour (Bowmaker 2008). Cues or signals of airborne molecules are received by chemoreceptors in the olfactory epithelium. Anurans also possess a vomeronasal organ (Eisthen 2000) proposed to only detect non-volatile molecules (Wysocki et al. 1980; Halpern and Martinez-Marcos 2003), whereas recent studies in *Mus musculus* (Muroi et al. 2006) and *Bufo bufo* (Merkel-Harff and Ewert 1991) suggest otherwise.

In reality, several anuran species represent an exception to our extremely abridged and generalized outline of sensory transducers. A recent study for example shows that the Malagasy frog *Mantidactylus betsileanus* possesses a unique intranasal anatomy (Junk et al. 2014), which might be a development related to the frog’s pheromone-producing femoral glands (Poth et al. 2012). There is also increasing evidence that many anurans have surprisingly good night vision (Cummings et al. 2008; Taylor et al. 2008; Gomez et al. 2009, 2010). *Hyla arborea* females were shown to discriminate between differently coloured male throats under nighttime light conditions, but it remains unsure, if the discrimination is based on differences in achromatic signals or if anurans are actually capable of distinguishing colours at night (but see Hailman and Jaeger 1974; Gomez et al. 2009).

Very important aspects to bear in mind when considering sexually selected traits, are not only the sensory properties of the receiver, but the actual signal components receivers respond to. In regions where *A. femoralis* calls partially overlapped with the co-occurring frog *Ameerega trivittata*, receivers of *A. femoralis* show no phonotactic reactions within the frequency range found in the call spectrum of both species (Amézquita et al. 2006). Thus, in case of sound interference by abiotic or biotic noise, it is not necessarily the signal that needs to be adapted to improve the signal-to-noise ratio. To understand the anuran sensory world in the light of sexual selection as a cause for signal adaptations, the coupled evolution of signals, sensory system, signalling behaviour and habitat choice have to be taken into account (sensory drive hypothesis) (Endler 1992; Endler and Basolo 1998). Anuran signalling traits and behaviours are additionally affected by perceptual biases in female preference and male–male interaction particularly in regard to species recognition and predator avoidance (Ryan and Keddy-Hector 1992; reviewed in Ryan and Cummings 2013). Finally, to understand driving forces and constraints of signal design and behaviour, regardless of their uni- or multimodality, we have to investigate perceptual and cognitive mechanisms of signal processing (see Hoke et al. 2005; reviewed in Miller and Bee 2012).

## Conclusion and outlook

“Until recently, efforts at understanding chorus interactions have been limited to recordings of interactions occurring over relatively small spatial scales involving just a few individuals (e.g. dyadic or triadic interactions among neighbours). Recording interactions over large spatial (and also temporal) scales was too technologically challenging, labour intensive, or both. New technological advances promise to change all this by enabling researchers to explore the complexity of chorus organization in ways only imagined in the late 1970s” (Bee et al. 2013).

As aforementioned, similar technical and conceptual advances in studies on visual and olfactory signals start to promote the research field (Cummings 2004, 2007; also see Ramsey et al. 2011). Answers to questions so elaborately proposed by Wells (1977) in the light of vocal signalling activity during social behaviour and behavioural ecology have filled textbooks (Ryan 1985; Gerhardt and Huber 2002; Narins et al. 2007) and further inspired groundbreaking research on acoustic communication systems (reviewed in Bee et al. 2013) leading from the “matched filter hypothesis” (Capranica and Moffat 1983; Gerhardt and Schwarz 2001) to the use of robotic frogs to study signal function (e.g. Narins et al. 2003; Klein et al. 2012). Today’s biological research has started to look at the big picture and integrates several sensory modalities, communication behaviour, environmental influences, perceptual biases and mechanisms of the species under investigation. Recent theoretical studies (Partan and Marler 1999, 2005; Hebets 2011) suggest different hypotheses to explain the function of signals in multimodal communication systems: multimodal or multiple signal components may evolve when they increase the signal content (content-based hypothesis), facilitate the perception of each other (inter-signal interaction hypothesis), or enhance signal transmission for instance in noisy environments (efficacy-based hypothesis) (Hebets and Papaj 2005). Based on their assumed information content, composite signals (Partan and Marler 2005) that occur together, can be further classified as redundant (all signal components elicit an equivalent response in the receiver) or non-redundant (signal components elicit a different response in the receiver). Anuran amphibians are excellent model species to test receiver responses, e.g. via female phonotaxis or male aggressive behaviour, to stimuli in uni- and multimodal playback studies and thereby help to better understand the function of signals in relation to the proposed hypothesis. Recent investigations of anuran communication systems on isolated and combined signal components across sensory modalities also allow suggestions about perceptual processes influencing signal evolution and underlying mechanisms (Taylor and Ryan 2013).

### Challenges in investigating multimodal signalling

The degree to which individual and/or combined components influence receivers in multimodal communication still remains difficult to generalize across species (Taylor et al. 2011b; Preininger et al. 2013b). To understand the efficacy of signals presented in two or more sensory modalities it is important to study the conditions in which they are presented. The efficiency of signal transmission and its effectiveness in modifying the behaviour of a receiver (Endler 2000) constitute the basis for research on signal design and evolution. Furthermore, neurobiological studies could shed much needed light on how the frog brain integrates multimodal signals (Hoke et al. 2004, 2005; Chakraborty et al. 2010; see Miller and Bee 2012; Taylor and Ryan 2013). Investigations should continue to focus on the environmental conditions which potentially favour a signal modality or their interaction and sensory modality differentiations due to detection, transmission and reception (e.g. Hoke et al. 2004). Further studies should also take into account the signal orientation of single and combined components towards same or differing receivers (e.g. mates or opponents). However, considering not only conspecific receivers but also heterospecific perceivers in the selection of signal design, we also emphasize the importance of investigations on the trade-off between efficacy and predation risk (Halfwerk et al. 2014).

We advocate that the natural history and habitat conditions of the respective study species are of utmost importance for understanding the evolution of signal design. Similarities in communication strategies do not necessarily indicate the same underlying mechanism, but could be of convergent origin and could have developed under related or different selection pressures. Interactions between sender and receiver under natural conditions could eventually help to understand the success of a certain signal and modality in the sense of signal efficacy and sensory drive.
